# Perinatal outcome and timing of selective fetal reduction in dichorionic diamniotic twin pregnancies: a single-center retrospective study

**DOI:** 10.3389/fmed.2023.1327191

**Published:** 2024-01-16

**Authors:** Gang Zou, Qingfang Ji, Jianping Chen, Luye Zhang, Qianqian Sun, Yaqi Shi, Yingjun Yang, Fenhe Zhou, Xing Wei, Luming Sun

**Affiliations:** Shanghai Key Laboratory of Maternal Fetal Medicine, Department of Fetal Medicine and Prenatal Diagnosis Center, School of Medicine, Shanghai Institute of Maternal-Fetal Medicine and Gynecologic Oncology, Shanghai First Maternity and Infant Hospital, Tongji University, Shanghai, China

**Keywords:** timing of selective reduction, gestational age at surgery, perinatal outcomes, gestational age at delivery, preterm birth, dichorionic diamniotic twin pregnancies

## Abstract

**Objective:**

The study aimed to evaluate the pregnancy outcomes of dichorionic diamniotic twin pregnancies that were reduced to singletons at different gestational ages.

**Study design:**

This was a retrospective cohort study of twin pregnancies that underwent fetal reduction to singletons in a single tertiary referral center between 2011 and 2020. A total of 433 cases were included. The cohort was divided into five groups according to gestational age at surgery: Group A: <16 weeks (125 cases); Group B: 16–19^+6^ weeks (80 cases); Group C: 20–23^+6^ weeks (74 cases); Group D: 24–26^+6^ weeks (48 cases); and Group E: ≥27 weeks (106 cases). Outcome data were obtained by reviewing the electronic medical records or interviews.

**Results:**

Selective reduction was technically successful. The clinical characteristics of the population were not different. The overall live birth rate and the survival rate were 96.5 and 95.4%, respectively. Although the rate of spontaneous miscarriage was comparable, gestational age at delivery significantly differed among groups (*p* < 0.001). Additionally, there was a trend that gestational age at delivery decreased with the increasing gestational age at surgery in Groups A, B, C, and D, whereas gestational age at delivery in Group E was later than that in Group D. In Groups A, B, C, and D, the rates of preterm birth at <32 weeks and <34 weeks increased with the increasing gestational age at surgery, while the rates in Group E were significantly lower than that in Group D. Regression analysis showed that timing of reduction may be an independent factor after adjusting for maternal age, parity, pre-pregnancy BMI, ART, and cervical length.

**Conclusion:**

Selective reduction performed by experienced hands for a dizygotic abnormal twin is safe and effective. Gestational age at surgery (<26^+6^ weeks) was inversely correlated with gestational age at delivery and positively with the rate of preterm birth. Reduction after 27 weeks, where legal, can be performed with a good outcome for the retained fetus.

## Introduction

Owing to the ubiquitous deployment of assisted reproductive technologies (ART) in clinical practice and the growing trend toward pregnancy at an advanced maternal age, the incidence of twin/multiple gestations has markedly surged in recent decades (1, 2). Twin/multiple gestations are frequently linked to a plethora of obstetrical complications for both the mother and the fetus, including, but are not limited to, premature delivery, low birth weight, preeclampsia, anemia, postpartum hemorrhage, intrauterine growth restriction, and severe neonatal morbidity (3). Notably, the risk of preterm birth in multiple pregnancies is significantly higher than in singleton pregnancies, with an odds ratio of up to sixfold (4). The occurrence of preterm birth accounts for up to 75% of all perinatal complications and is causally associated with more than 50% of long-term maternal-fetal morbidity (5). While many preterm infants may survive, they are at elevated risk for developing neurological impairments as well as respiratory and gastrointestinal complications (6).

Fetal reduction techniques have been employed in multiple pregnancies to mitigate the risk of unfavorable perinatal outcomes, thereby promoting optimal obstetrical outcomes. Although fetal reduction may be suggested for twin pregnancies with iatrogenic abnormalities, it is not typically advocated for non-complicated twin pregnancies. Nevertheless, twin and multiple gestations are associated with an increased incidence of structural and chromosomal anomalies compared with singleton pregnancies (7), and selective fetal reduction may be considered an alternative to induced abortion for twin gestations manifesting with one or more abnormal fetuses while preserving the unaffected fetus. Studies have shown that selective reduction of abnormal fetuses in multiple pregnancies leads to a better pregnancy outcome but may increase the risk of pregnancy loss or preterm birth (8–10). Several studies have investigated the association between the timing of selective fetal reduction and perinatal outcomes (11, 12); however, the findings were inconsistent.

The present study aimed to determine the effect of the timing of selective reduction, in more detail, on the overall pregnancy outcomes, most importantly early delivery, in a larger retrospective cohort of dichorionic diamniotic twin pregnancies (DCDA).

## Materials and methods

### Study participants

This was a retrospective cohort study of dichorionic diamniotic twin gestations with a prospective design. We assessed all 458 pregnant women who underwent fetal reduction between 2011 and 2020 at the Department of Fetal Medicine in the First Maternal and Infant Health Hospital affiliated with Tongji University. Among them, 24 patients were lost to follow-up, with a rate of loss to follow-up of 5.2%. One patient underwent induced abortion at 24^+2^ weeks due to malformation found in the non-reduced twin after selective reduction. Finally, 433 patients were included in the present study.

### Procedure

All patients underwent clinical, ultrasonographic, and genetic evaluation as well as clinical counseling, at the Department of Fetal Medicine before surgery. A multidisciplinary evaluation was carried out when necessary, and ethical approval was obtained for those who reduced after gestational age (GA) of 28 weeks. The ethical approval process consists of three steps: (1) an application of selective reduction filled by the couple would be submitted to the ethics committee of the hospital; (2) the detail diagnoses, reports, and suggestions from doctors would be provided; and (3) the ethics committee would have a discussion on the application. The two main principles considered by the ethics committee are maternal safety and a clear poor prognosis for the fetus. Preoperative communication was conducted, an individualized fetal reduction scheme was formulated under the premise of respecting the wishes of patients and their families, and informed consent was signed by the patients and their families. In all cases, the chorionicity was verified by the ultrasound scan record in early pregnancy, and the target fetus was positioned by means of fetal sex, fetal structural abnormalities, placental location, and soft markers. Under the guidance of ultrasound, a 21G puncture needle was inserted through the abdominal wall of the patient into the fetal heart. Blood from the fetal heart was extracted, with 2 mL reserved for chromosome karyotype analysis. Following the injection of 2–6 mL of a 10% KCL solution, the fetal heart condition was observed to confirm cardiac arrest before withdrawing the puncture needle. Subsequent to the fetal reduction, an ultrasound scan was used to verify the cardiac arrest of the reduced fetus and assess the heart rate of the remaining fetus.

### Data collection and outcome measures

Clinical data was collected by reviewing the electronic medical records, and outcome measures were collected from medical records or direct phone interviews with the women. GA at surgery, birth weight, Apgar score, mode of delivery, the rate of spontaneous miscarriage (fetal death prior to 28 weeks), GA at delivery, pregnancy complications, including hypertension disease of pregnancy, gestational diabetes were determined in each group. The primary outcomes were GA at delivery, delivery prior to 32 weeks and 34 weeks, as well as preterm birth (a live birth after 28 weeks of gestation but less than 37 weeks).

### Statistical analysis

SAS 9.4 (SAS Institute Inc.) was used for statistical analysis. Normality of the data distribution was tested using the Kolmogorov–Smirnov tests. The study cohort was divided into five groups according to the GA at surgery: Group A: <16 weeks (125 cases); Group B: 16–19^+6^ weeks (80 cases); Group C: 20–23^+6^ weeks (74 cases); Group D: 24–26^+6^ weeks (48 cases); and Group E: ≥27 weeks (106 cases). For continuous variables, mean and standard deviation (X ± SD) were used for those with normal distribution, and analysis of variance (ANOVA) was used for comparison among groups; median (inter-quartile range, IQR) was used for those with non-normal distribution, and the Kruskal–Wallis test was used for comparison among groups. Categorical data were presented as number and proportion/ percentage, and comparison between groups was performed using the Chi-square test or Fisher’s exact test as appropriate. A Kaplan–Meier curve was constructed to demonstrate the GA at delivery stratified by the GA at surgery, in which live birth was set as the event and others were set as censor. Logistic regression analysis was used to determine whether the timing of selective fetal reduction was associated with early delivery after adjustment for maternal age, parity, pre-pregnancy BMI (body mass index, kg/m^2^), conception method, and cervical length. The significance threshold was set as *p* < 0.05.

## Results

### Clinical characteristics of the study population

From 2011 to 2020, a total of 458 DCDA patients underwent selective fetal reduction with potassium chloride at the Department of Fetal Medicine, the First Maternity and Infant Hospital affiliated with Tongji University. Of these, 24 cases were lost to follow-up (5.2%), and one case underwent induced abortion due to malformation of the reserved fetus after surgery. Finally, 433 patients were included in the present study, with malformation accounted for about 95% of the surgical indication. The average GA at surgery was 20.7 (15.6, 26.9) weeks, of which 125 cases (28.9%) received selective fetal reduction at GA of <16 weeks (Group A), 80 cases (18.5%) at 16–19^+6^ weeks (Group B), 74 cases (17.1%) at 20–23^+6^ weeks (Group C), 48 cases (11.1%) at 24–26^+6^ weeks (Group D), and 106 cases (24.5%) at GA of ≥27 weeks (Group E), including 13 cases were reduced at GA of ≥28 weeks for major malformations (9 cases with severe genetic disease, and 4 cases with major structural abnormalities), which were diagnosed after 28 weeks, with ethical approval (Supplementary Table S1).

Table 1 displays the clinical characteristics of the study cohort. The average maternal age and pre-pregnancy body mass index (BMI) for all cases were 31.4 ± 4.5 years and 22.0 ± 3.2 kg/m^2^, 77.8% of the cases were nulliparous, 74% conceived by ART, 50.8% were with anterior placenta, and all of which were not different among the five groups. However, the average cervical lengths were shorter in Groups D and E (32.4 ± 9.5 mm and 31.5 ± 8.2 mm, respectively) than in Groups A, B, and C (35.3 ± 5.8 mm, 37.0 ± 5.7 mm, and 36.8 ± 6.1 mm, respectively, *p* < 0.001), corresponding to higher proportions of shorter cervical length (<25 mm or 28 mm) in the former two groups. In addition, the complications of hypertension/preeclampsia and diabetes/GDM were not different among groups.

**Table 1:** Baseline characteristics according to different GA at reduction.

Parameters	Group A <16w (*n* = 125)	Group B 16–19^+6^w (*n* = 80)	Group C 20–23^+6^w (*n* = 74)	Group D 24–26^+6^w (*n* = 48)	Group E ≥27w (*n* = 106)	Total (*n* = 433)	*p*
**Age (years)**	31.2 ± 4.3	31.2 ± 4.0	31.4 ± 5.3	31.7 ± 5.2	31.8 ± 4.2	31.4 ± 4.5	0.8310
**BMI (kg/m** ^**2**^ **)**	21.9 ± 3.1	22.0 ± 4.0	22.4 ± 3.2	22.1 ± 3.0	21.5 ± 2.8	22.0 ± 3.2	0.4660
**Parity**							0.7440
Nulliparous	102 (81.6)	63 (78.8)	55 (74.3)	37 (77.1)	80 (75.5)	337 (77.8)	
Parous	23 (18.4)	17 (21.2)	19 (25.7)	11 (22.9)	26 (24.5)	96 (22.2)	
**Conception method**							
Spontaneous	26 (20.8)	19 (23.8)	23 (31.1)	10 (20.8)	35 (33.0)	113 (26.1)	
ART	99 (79.2)	61 (76.2)	51 (68.9)	38 (79.2)	71 (67.0)	320 (74.0)	
**Placenta location**							
Anterior	68 (54.4)	38 (47.5)	30 (40.5)	25 (52.1)	59 (55.7)	220 (50.8)	0.2730
Non-anterior	57 (45.6)	42 (52.5)	44 (59.5)	23 (47.9)	47 (44.3)	213 (49.2)	
**Cervical length at surgery(mm)**	35.3 ± 5.8	37.0 ± 5.7	36.8 ± 6.1	32.4 ± 9.5	31.5 ± 8.2	34.6 ± 7.3	<0.001
<25	1 (0.8)	0 (0.0)	2 (2.7)	8 (16.7)	16 (15.1)	27 (6.2)	<0.001^F^
<28	7 (5.6)	4 (5.0)	3 (4.1)	10 (20.8)	28 (26.4)	52 (12.0)	<0.001
**Hypertension/preeclampsia**	5 (4.0)	1 (1.3)	2 (2.7)	1 (2.1)	3 (2.8)	12 (2.8)	0.837^F^
**Diabetes/GDM**	6 (4.8)	4 (5.0)	4 (5.4)	0 (0.0)	9 (8.5)	23 (5.3)	0.300^F^

^F^, Fisher’s exact test; BMI, body mass index.

### Perinatal outcomes

The perinatal outcomes are presented in Table 2. Spontaneous miscarriage (<28 weeks) happened in 12 cases (2.8%), and termination of pregnancy (TOP) due to intrauterine fetal death (IUFD)/ unfavorable fetal condition in 3 cases (0.7%), corresponding to a live birth rate of up to 96.5% for the whole cohort. The overall pregnancy outcomes differed among the five groups, with Groups B and C of a little lower live birth rates (92.5 and 94.6%, *p* = 0.045). In contrast, the rates of spontaneous miscarriage <28 weeks or within 2 weeks after surgery and fetal loss <28 weeks were a little higher in Groups B and C (marginal significant, *p* value ranging from 0.052 to 0.072). Importantly, no fetal loss occurred after 28 weeks, and the rates of spontaneous miscarriage <24 weeks or fetal loss were not different among groups.

**Table 2:** Perinatal outcomes according to different GA at reduction.

Outcomes	Group A <16w (*n* = 125)	Group B 16–19^+6^w (*n* = 80)	Group C 20–23^+6^w (*n* = 74)	Group D 24–26^+6^w (*n* = 48)	Group E ≥27w (*n* = 106)	Total (*n* = 433)	*p*
**Overall pregnancy outcomes**							0.0448^F^
**Live birth**	121 (96.8)	74 (92.5)	70 (94.6)	48 (100.0)	105 (99.1)	418 (96.5)	
**Spontaneous miscarriage**	4 (3.2)	4 (5.0)	4 (5.41)	0 (0)	0 (0)	12 (2.8)	
**IUFD/TOP due to unfavorable fetal condition**	0 (0.0)	2 (2.5)	0 (0.0)	0 (0.0)	1 (0.94)	3 (0.7)	
**Spontaneous miscarriage (weeks)**							
<24	1 (0.8)	2 (2.5)	2 (2.7)	0 (0.0)	0 (0.0)	5 (1.2)	0.2666^F^
<28	4 (3.2)	4 (5.0)	4 (5.4)	0 (0.0)	0 (0.0)	12 (2.8)	0.0538^F^
**Within 2 weeks after surgery**	0 (0.0)	1 (1.3)	2 (2.7)	0 (0.0)	0 (0.0)	3 (0.7)	0.0959^F^
**Within 2 weeks after surgery**	0 (0.0)	2 (2.5)	2 (2.7)	0 (0.0)	0 (0.0)	4 (0.9)	0.0526^F^
**Fetal loss (weeks)** ^**a**^							
<24	1 (0.8)	3 (3.8)	2 (2.7)	0 (0.0)	0 (0.0)	6 (1.4)	0.1314^F^
<28	4 (3.2)	6 (7.5)	4 (5.4)	0 (0.0)	1 (0.9)	15 (3.5)	0.0718^F^
**GA at delivery** ^b^**(weeks)**	37.9 ± 3.3	36.6 ± 4.9	36.1 ± 4.5	34.7 ± 3.9	35.9 ± 3.0	36.5 ± 4.0	<0.0001
<28	6 (4.8)	5 (6.3)	6 (8.1)	0 (0.0)	0 (0.0)	17 (3.9)	0.0085^F^
<32	9 (7.2)	7 (8.8)	14 (18.9)	15 (31.2)	11 (10.4)	56 (12.9)	0.0002
<34	10 (8.0)	11 (13.8)	17 (23.0)	20 (41.7)	21 (19.8)	79 (18.2)	<0.0001
**Within 2 weeks after surgery**	0 (0.0)	2 (2.5)	2 (2.7)	0 (0.0)	8 (7.6)	12 (2.8)	0.0057^F^
**Interval between surgery and delivery**	24 (22.9, 25)	20.4 (18.8, 21.9)	15.3 (12.1, 17.1)	9.8 (5.8, 13.1)	8.5 (5.6, 10.3)	16.0 (9.1, 22.6)	<0.0001^kw^
**Number of live births**	*n* = 121	*n* = 74	*n* = 70	*n* = 48	*n* = 105	*n* = 418	
**Preterm birth (weeks)**							
<28	2 (1.7)	1 (1.4)	2 (2.9)	0 (0.0)	0 (0.0)	5 (1.2)	0.4117^F^
<32	5 (4.1)	3 (4.1)	10 (14.3)	15 (31.3)	11 (10.5)	44 (10.5)	<0.0001
<34	6 (5.0)	7 (9.5)	13 (18.6)	20 (41.7)	21 (20.0)	67 (16.0)	<0.0001
**Within 2 weeks after surgery**	0 (0.0)	0 (0.0)	0 (0.0)	0 (0.0)	8 (7.6)	8 (1.9)	0.0001^F^
**Mode of delivery**							0.4678
Vaginal delivery	47 (38.8)	21 (28.4)	23 (32.9)	15 (31.3)	30 (28.6)	136 (32.5)	
C-section	74 (61.2)	53 (71.6)	47 (67.1)	33 (68.7)	75 (71.4)	282 (67.5)	
**Birth weight(g)**	3099.6 ± 611.7	3048.8 ± 605.7	2791.6 ± 749.6	2385.1 ± 819	2,556 ± 681.4	2818.5 ± 726.6	<0.0001
**1-min Apgar score < 7**	1 (0.8)	5 (6.8)	7 (10.0)	4 (8.3)	10 (9.5)	27 (6.5)	0.0112^F^
**5-min Apgar score < 7**	0 (0)	1 (1.4)	1 (1.4)	0 (0)	2 (1.9)	4 (1.0)	0.4980^F^
**NICU hospitalization**	17 (14.1)	16 (21.6)	24 (34.3)	25 (52.0)	41 (39.0)	123 (29.4)	<0.0001
**Neonatal respiratory distress syndrome (NRDS)**	3 (2.5)	5 (6.8)	7 (10.0)	10 (20.8)	8 (7.6)	33 (7.9)	0.0024
**Neonatal death**	1 (0.8)	2 (2.7)	1 (1.4)	1 (2.1)	0 (0.0)	5 (1.2)	0.3992^F^
**Overall survival rate at 28 days after birth**	120 (96.0)	72 (90.0)	69 (93.2)	47 (97.9)	105 (99.1)	413 (95.4)	0.0363^F^

GA, gestational age.

a Fetal loss includes spontaneous miscarriage and IUFD/TOP due to unfavorable fetal condition.

b GA at delivery for all cases including live birth, spontaneous miscarriage, IUFD/TOP due to unfavorable fetal condition.

NRDS: Neonatal respiratory distress syndrome; ^F^: Fisher exact test; ^kw^: Kruskal–Wallis test.

**Figure 1:**
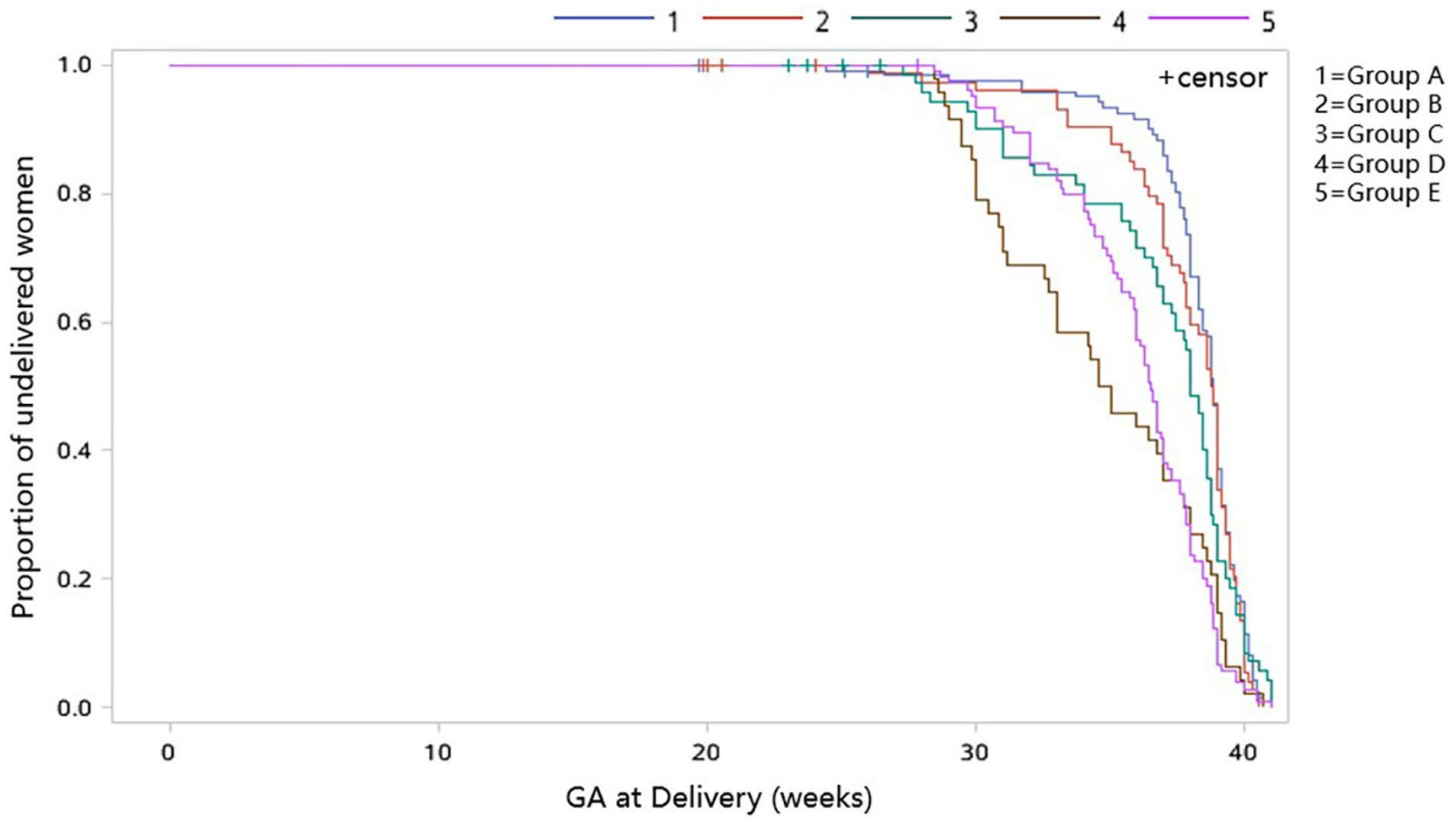
Kaplan–Meier curve showing the GA at delivery for all five groups.

The mean GA at delivery (including spontaneous miscarriage, TOP, and live birth) was 36.5 ± 4.0 weeks for the cohort. The GA at delivery differed significantly between groups (*p* < 0.001), and there was a trend that the GA at delivery decreased with the increasing GA at surgery in Groups A, B, C, and D (37.9 ± 3.3 weeks, 36.6 ± 4.9 weeks, 36.1 ± 4.5 weeks, and 34.7 ± 3.9 weeks, respectively), whereas the GA at delivery in Group E (35.9 ± 3.0 weeks) was later than that in Group D (Table 2), which could be clearly seen in the Kaplan–Meier curve showing the GA at delivery (Figure 1). The rates of delivery <28 weeks were 4.8, 6.3, and 8.1% in Groups A, B, and C; however, it did not happen in Groups D and E (*p* = 0.0085). In Groups A, B, C, and D, the rates of delivery at <32 weeks (7.2, 8.8, 18.9, and 31.2%, respectively) and < 34 weeks (8.0, 13.8, 23.0, and 41.7%, respectively) increased with the increasing GA at surgery, while the rates in Group E (<32 weeks: 10.4%; <34 weeks: 19.8%) were significantly lower than that in Group D, even when those who received surgery after 32 weeks in Group E were excluded (<32 weeks: 11.1%; <34 weeks: 21.2%). The rate of delivery within 2 weeks after surgery in Group E was 7.6%, which was highest (*p* = 0.0057). Moreover, the interval between surgery and delivery decreased with increasing GA at surgery (*p* < 0.0001^kw^). Similarly, for those with live birth, the rate of preterm birth <32 weeks (*p* < 0.0001), <34 weeks (*p* < 0.0001), and within 2 weeks after surgery (*p* < 0.0001^F^) among the five groups were significantly different, and the trend is identical to the outcome of delivery <32 weeks, <34 weeks, and within 2 weeks after surgery for the whole cohort. No significant difference was found in the mode of delivery (*p* = 0.4678). However, the birth weight, rate of NICU hospitalization, and neonatal respiratory distress syndrome (NRDS) were different among the five groups (all *p* value < 0.05), with Group D having the lowest birth weight, the highest rate of NICU hospitalization, and NRDS, and the trend was in accordance with GA at delivery. There was no difference in the 5-min Apgar score among the five groups. A total of five neonatal deaths were observed in the whole cohort (1.2%), and no difference was found in neonatal mortality among different groups (*p* = 0.3992^F^). The overall survival rate at 28 days after birth was 95.4%, with the lowest survival rate of 90.0% in Group B, followed by Group C of 93.2%, Group A of 96.0%, Group D of 97.9%, and Group E of 99.1% (*p* = 0.0363^F^).

### Regression analysis

After adjusting for maternal age, parity, pre-pregnancy BMI, ART, and cervical length, compared with Group A, Groups C and D were at increased risk of delivery <32 weeks and < 34 weeks. The odds ratios (ORs) for delivery <32 weeks were 3.39 (95% confidence interval, CI:1.32, 8.73) and 4.63 (95% CI:1.75, 12.23), respectively, and the ORs for delivery <34 weeks were 3.78 (95% CI:1.58, 9.06) and 7.17 (95% CI, 2.92, 17.6), respectively. However, the risks of early delivery were not significant in Groups B and E (Table 3).

**Table 3:** Timing of fetal reduction and early delivery.

GA at surgery	Delivery prior to 32 weeks		Delivery prior to 34 weeks	
	cOR (95% CI)	aOR (95% CI)*	cOR (95% CI)	aOR (95% CI)*
Group A (<16w)	Ref	Ref	Ref	Ref
Group B (16–19^+6^w)	1.24 (0.44, 3.46)	1.43 (0.49, 4.19)	1.83 (0.74, 4.54)	2.09 (0.82, 5.33)
Group C (20–23^+6^w)	3.01 (1.23, 7.35)	3.39 (1.32, 8.73)	3.43 (1.48, 7.97)	3.78 (1.58, 9.06)
Group D (24–26^+6^w)	5.86 (2.35, 14.59)	4.63 (1.75, 12.23)	8.21 (3.46, 19.49)	7.17 (2.92, 17.6)
Group E (≥27w)	1.49 (0.59, 3.75)	0.95 (0.34, 2.64)	2.84 (1.27, 6.34)	1.98 (0.84, 4.69)

*Adjusted for age, parity, pre-pregnancy BMI, ART, and cervical length.

## Discussion

### Principal findings

The retrospective cohort study indicated that selective fetal reduction is safe for dichorionic diamniotic twin pregnancies, with an overall live birth rate of 96.5% and a survival rate of 95.4%. The rates of spontaneous miscarriage <28 weeks or within 2 weeks after surgery, as well as fetal loss <28 weeks, were a little higher for those undergoing selective reduction at GA of 16–19^+6^ weeks and 20–23^+6^ weeks, resulting in lower rates of live birth and overall survival. For our study cohort, the significant difference about the overall pregnancy outcomes was found between the five groups. The rates of spontaneous miscarriage are comparable among different groups, while the GA of delivery significantly differed. When the surgery was conducted <27 weeks, the rates of delivery <32 weeks and < 34 weeks increased with increasing GA at surgery, which were highest in those undergoing surgery at 24–26^+6^ weeks, followed by those undergoing surgery at 20–23^+6^ weeks. However, for those undergoing surgery ≥27 weeks, the average GA at delivery was later, and the rate of early delivery (<32 and <34 weeks) was lower than for those who underwent surgery at 24–26^+6^ weeks. Multivariate analysis revealed that, compared with those who underwent surgery <16 weeks, those undergoing surgery at 20–23^+6^ weeks and 24–26^+6^ weeks were at increased risk of delivery <32 weeks and <34 weeks after adjusting for maternal age, parity, pre-pregnancy BMI, ART, and cervical length. Although having a higher risk of early delivery, those undergoing surgery at 24–26^+6^ weeks also attained a high overall survival rate of 98%.

## Results

Generally, the results indicated that selective reduction at 20–23^+6^ weeks and 24–26^+6^ weeks leads to a higher rate of early delivery (<32 or <34 weeks), but those undergoing surgery ≥27 weeks result in later GA at delivery and support the ISUOG guidelines, which recommend performing selective reduction preferably in the first trimester or opt for late reduction in the third trimester when diagnosis is made in the second trimester (if the law permits) (13). In addition, recent research has indicated that compared with late first-trimester reduction of twins (11–14 weeks), second-trimester reduction (15–23 weeks) is associated with an increased rate of prematurity and adverse neonatal outcomes (12).

### Clinical implications

We noticed that several studies are divided referring to the relationship between GA of selective reduction and the risk of preterm birth and fetal loss (11, 14–17). In many studies, the higher frequency of preterm birth was more common along with the later timing of selective reduction. Bennasar et al. found that those undergoing selective reduction at 18–23^+6^ weeks had higher risk of a pregnancy loss rate (12% vs. 3.1%), preterm delivery (pregnancy loss not included) <28 (9.1% vs. 6.3%) and <32 weeks (18.2% vs. 9.5%) in comparison with those undergoing surgery <18 weeks. A multiple-center study by Evans et al. (18) reported that the fetal loss rates (<24 weeks, 7.1%; all loss, 7.9%) were not different by GA at surgery (9–12 weeks, 5.4%; 13–18 weeks, 8.7%; 19–24 weeks, 6.8%; and ≥ 25 weeks, 9.1%) in 402 selective reduction dizygotic twins (18). Another most recent systematic review by Sorrenti et al. included 649 dichorionic twin pregnancies and found that the risk of fetal loss prior to 24 weeks (1% vs. 8%, odds ratios, OR = 0.25), preterm birth <37 (19% vs. 45%, OR = 0.36), <34 (4% vs. 19%, OR = 0.24), and <32 (3% vs. 20%, OR = 0.21) weeks for those undergoing selective reduction before 18 weeks were, respectively, significantly lower than those after 18 weeks (19). Zemet et al. (12) found in a cohort of 248 fetal reduction cases that those undergoing fetal reduction at 11–14 weeks had lower rates of pregnancy loss (0.6% vs. 1.3%), preterm delivery (pregnancy loss not included) <32 weeks (1.8% vs. 8.0%) and <34 weeks (1.8% vs. 12.0%) than those undergoing fetal reduction at approximately 15–23 weeks, though they found that the GA at delivery was not different from the GA at surgery (12). Another most recent study of 172 selective reduction cases, Kristensen et al. reported that those undergoing surgery before 14 weeks had a lower rate of adverse pregnancy outcome (miscarriage, stillbirth), preterm birth <28 and <32 weeks (pregnancy loss not included), compared with those reduced after 14 weeks (1.4% vs. 6.1%; 0% vs. 4.3%; 2.8% vs. 5.4%, respectively) (20). The loss rate and preterm delivery rates from the above-mentioned studies were comparable to those in our study; however, the preterm delivery rate in the study of Zemet and Kristensen was lower than the result of this study. The difference mainly originates from the different GA at surgery; in this study, about one-third of the cases were reduced after 24 weeks, which is distinguishing from others. Moreover, most of the studies included twins with an abnormal fetus, whereas the study of Zemet included cases undergoing multifetal pregnancy reduction. Furthermore, our study showed that there was a different outcome of overall survival rate at 28 days after birth, and no significant difference was found in fetal loss among each group. The findings were not consistent with some studies (15, 17, 21), suggesting that the GA of selective reduction may not correlate with the rate of fetal loss. A firm conclusion needs more studies.

### Research implications

In the current study, we investigated the effect of timing of selective reduction for dichorionic diamniotic twin pregnancies on pregnancy outcomes, mainly on early delivery. It was noteworthy that significant differences in cervical length between groups were displayed (all *p* value < 0.0001). Previous studies have confirmed an inverse relation between the cervical length and the frequency of preterm delivery (22), which may predict intra-amniotic inflammation as well as preterm delivery (23). At 24 weeks’ gestation, for example, a cervical length of <22 mm was associated with about 20% risk of preterm delivery (22). Our results are similar to the previous studies focusing on the relationship between cervical length and the incidence of preterm birth (24–27). Furthermore, the later timing of selective reduction, the higher the rate of preterm birth prior to 34 weeks of gestation. Since Group E may include patients with selective reduction in early third trimester, the preterm birth rate did not strictly follow this above-mentioned rule. However, the fact was clear that there was a significant increase in preterm birth within 2 weeks after surgery in Group E. Moreover, we assumed that the occurrence of preterm birth may also be related to other factors, such as the family and social environment of the patients and the intrauterine localization of the reduced fetus. Larger prospective multi-center trials determining the pregnancy outcomes by GA at selective reduction are needed to ascertain the results.

### Strength and limitations

This study noteworthy involving the largest cohort to investigate pregnancy outcomes by different gestational ages of selective reduction. This approach enables us to investigate the effect of GA at surgery in more detail and may provide preoperative counseling and advice to DCDA patients with clear indications for selective reduction. All procedures were performed by the same experienced team, thus eliminating significant bias in the procedures itself. However, several limitations should be acknowledged. Firstly, this is a retrospective cohort study, though with a prospective study design, it is susceptible to potential confounding factors, especially loss to follow-up. In addition, a part of information was collected via phone interviews with patients, which might be susceptible to recall bias.

## Conclusion

In conclusion, selective reduction by potassium chloride is safe for diamniotic twin pregnancies with iatrogenic abnormalities; it can attain an overall survival rate of more than 95%. Overall, those undergoing surgery at <16 weeks could obtain a lower fetal loss and preterm delivery rate. The risk of delivery <32 weeks and <34 weeks increased in those undergoing surgery at 24–26^+6^ and 20–23^+6^ weeks after adjusting for potential confounders in comparison with those at <16 weeks. In contrast, the rate of early delivery (<32 and <34 weeks) was lower in those undergoing surgery ≥27 weeks than those who had surgery at 24–26^+6^ weeks. Complete and exact fetal assessments by ultrasonic examination, chorionic puncture, and amniocentesis during the first 3 months of pregnancy are important, which may allow the selective reduction before 16 weeks to obtain a better outcome. Notably, when the malformation is diagnosed in the late second trimester, selective reduction after 27 weeks or in early third trimester could be an option if the law permits.

## Data availability statement

The raw data supporting the conclusions of this article will be made available by the authors, without undue reservation.

## Ethics statement

Written informed consent was obtained from the individual(s) for the publication of any potentially identifiable images or data included in this article.

## Author contributions

GZ: Formal Analysis, Funding acquisition, Investigation, Supervision, Project administration, Writing – review & editing. QJ: Methodology, Investigation, Validation, Writing – original draft, Writing – review & editing. JC: Data curation, Investigation, Methodology, Writing – review & editing. LZ: Collection of the data. QS: Collection of the data. YS: Collection of the data. YY: Collection of the data. FZ: Collection of the data. XW: Collection of the data. LS: Investigation, Supervision, Visualization.
