# Supplementary material for: Perinatal outcome and timing of selective fetal reduction in dichorionic diamniotic twin pregnancies: a single-center retrospective study
Source: Front Med (Lausanne). 2024 Jan 16;10:1327191. doi: 10.3389/fmed.2023.1327191 (PMC10824961; doi:10.3389/fmed.2023.1327191)
Supplement: Supplementary file 1 [file Table_1.docx]

**Supplementary Table 1：Information of 13 case who underwent reduction beyond 28 weeks**

| **Case** | **age(year)** | **Conception method** | **Conditions for fetal reduction** | **GA at reduction（w）** |
| --- | --- | --- | --- | --- |
| Case1 | 36 | ART | Dandy-walker syndrome | 32.0 |
| Case2 | 34 | ART | Multiple deformity | 32.0 |
| Case3 | 31 | IART | FGR, chromosomal( 46,XN,dup(7)(p22p11) ) | 32.5 |
| Case4 | 28 | Spontaneous | Severe tetralogy of Fallot and Pulmonary atresia | 34.2 |
| Case5 | 25 | Spontaneous | Spina bifida | 31.6 |
| Case6 | 28 | ART | Cantrell syndrome | 32 |
| Case7 | 34 | ART | Trisomy 21 syndrome | 31.6 |
| Case8 | 34 | Spontaneous | Cardio-splenic Syndrome | 31.5 |
| Case9 | 32 | ART | Bilateral wide ventricles with subependymal hemorrhage | 33.2 |
| Case10 | 32 | ART | Cleft lip and palate and microcephaly | 30.5 |
| Case11 | 38 | ART | Tetralogy of Fallot and chromosomal disease(22q11.2 microdeletion） | 32.1 |
| Case12 | 32 | Spontaneous | Edema and maternal mirror syndrome | 30 |
| Case13 | 34 | ART | Chromosomal abnormality( 3q24q26.2 ) | 30.3 |
